# How Ingratiation Links to Counterproductive Work Behaviors: The Roles of Emotional Exhaustion and Power Distance Orientation

**DOI:** 10.3389/fpsyg.2020.02238

**Published:** 2020-09-01

**Authors:** Miao Yan, Yu-ping Xie, Jun Zhao, Yong-jun Zhang, Mohsin Bashir, Ying Liu

**Affiliations:** ^1^School of Management, Huazhong University of Science and Technology, Wuhan, China; ^2^School of Public Administration, Zhongnan University of Economics and Law, Wuhan, China; ^3^Business School, Henan University, Kaifeng, China; ^4^Lyallpur Business School, Government College University Faisalabad, Faisalabad, Pakistan

**Keywords:** ingratiation, emotional exhaustion, power distance orientation, counterproductive work behaviors, conservation of resources theory

## Abstract

Ingratiation is regarded as a powerful impression tactic that helps ingratiator achieve their intended goals. Although there is evidence that the consequences of ingratiation are not always positive, little research considers the dark effect of ingratiation on the ingratiator. Based on conservation of resources theory, we develop and test a model that links employees’ ingratiation to their counterproductive work behaviors. Data were collected from 216 supervisor-employee dyads. The results of examination with Mplus showed that ingratiation had a positive effect on counterproductive work behaviors, and emotional exhaustion played a mediating role in this relationship. Power distance orientation negatively moderated the relationship between ingratiation and emotional exhaustion and the indirect effect of emotional exhaustion on the relationship between ingratiation and counterproductive work behaviors. Our findings raise attention on the consequences of ingratiation for employees and the dark side of ingratiation for organization.

## Introduction

Ingratiation involves the deliberate using of flattery, enhancing others, or engaging in opinion conformity, in which an individual endorses the opinions held or espoused by another person to strengthen her relationship with the targeted individual (Tedeschi and Melburg, 1984; Higgins and Judge, 2004). Ingratiation can be focused in different directions, such as toward leaders or coworkers (Kipnis et al., 1980; Liden and Mitchell, 1988). Many scholars have become increasingly interested in the positive effects of ingratiation, which include favorable performance evaluations, promotions and higher exchange in relationships (Kumar and Beyerlein, 1991; Johnson et al., 2002; Lam et al., 2007). Westphal and Stern (2006) noted that ingratiation can be regarded as an act of submission or deference to another person that elicits goodwill and various forms of social support (Shropshire, 2010). A meta-analysis of 69 studies indicated that ingratiation was positively related to likeability and career success (Higgins et al., 2003). As a powerful impression tactic, ingratiation is used by employees to help them achieve their intended goals through a combination of affective and cognitive processes (Cooper, 2005).

However, the consequences of ingratiation are not always positive. There is considerable evidence that if individuals choose inappropriate tactics or incorrect timing, ingratiation will not produce the desired results (Aryee et al., 1996; Lam et al., 2007; Treadway et al., 2007). On the one hand, individuals who engage in ingratiation may be perceived as untruthful, unreliable and manipulative (Grant, 1996); thus, these individuals are unlikely to create favorable impressions and likely to experience low reward and low-quality reciprocal relationships (Lam et al., 2007). On the other hand, when the influence target is receptive to high levels of flattery and opinion conformity (Tedeschi and Melburg, 1984), the target may become overconfident in his judgment and capability and react inappropriately to strategic change, leading to a decline in organizational performance (Park et al., 2011). In addition, ingratiation may prompt ingratiators’ feeling of resentment toward the influence target due to the threat to their self-esteem (Rorty, 2000; Smith and Kim, 2007; Leach and Spears, 2008). Somewhat surprisingly, little research considers the dark effect of ingratiation on the ingratiator (Klotz et al., 2018). To address the lack of attention to this issue, the present study considers how employees’ ingratiation links to their subsequent counterproductive work behaviors.

Ingratiation is one of the most widely used influence tactics, and it may drain self-control resources (Vohs and Baumeister, 2004) because successful ingratiation requires the appearance of sincerity (Leary, 1995). Employees who experience depletion of self-control resources are more likely to steal and cheat (Gino et al., 2011). Moreover, employees are more likely to build resentment toward supervisors because ingratiation can threaten employees’ positive self-regard, which may trigger counterproductive work behaviors (Keeves et al., 2017; Klotz et al., 2018). It is noted that when the efforts of ingratiation unable to obtain rewards, employees will generate the perception of unfair treatment that may also lead to counterproductive work behaviors (Guglielmi et al., 2018).

Conservation of resources theory indicates that individuals strive to obtain, maintain and protect their resources. When the potential or real loss of these resources threatens them, individuals turn to other resources that offset the deleterious effects of this loss (Hobfoll, 1989). Ingratiation includes favor doing, opinion conformity, other-enhancement and self-presentation (Tedeschi and Melburg, 1984), and each of these behaviors may require employees to expend physical and psychological resources. The depletion of their resources makes employees feel stressed and threatened; this may lead to emotional exhaustion, which is a chronic state of emotional and physical fatigue that reflects employees’ sense of being depleted by their work (Maslach and Jackson, 1981; Maslach et al., 2001). If employees experience emotional exhaustion, they may overvalue the importance of withdrawal coping strategies (Leiter, 1993). Therefore, employees may conserve their resources and restore existing resource losses by engaging in counterproductive work behaviors (Spector et al., 2006).

Given that conservation of resources theory recognizes that certain personality traits have an effect on individuals’ reaction to the process of gaining resources and avoiding resource loss (Hobfoll et al., 1990; Koopman et al., 2016), we examine the moderating role of power distance orientation, which relates to employees’ values in relation to status, authority, and power in an organization (Kirkman et al., 2009). Employees with high power distance orientation tend to accept their top-down relationship with supervisors (Javidan et al., 2006) and regard ingratiation as a common workplace phenomenon; consequently, they may be less sensitive to resource loss from engaging in this behavior (Koopman et al., 2016). These employees should be less susceptible to emotional exhaustion and counterproductive work behaviors caused by ingratiation. We construct a moderated meditation model to test how and when ingratiation is linked to counterproductive work behaviors.

The theoretical perspective and empirical results of this study contribute to the literature in several ways. First, based on conservation of resources theory, this study explores the effect of ingratiation on counterproductive work behaviors to extend our understanding of the effect of ingratiation on the ingratiator (Park et al., 2011; Klotz et al., 2018). Second, this study examines the link between ingratiation and emotional exhaustion, revealing the mechanism of and theoretical explanation for the relationship between ingratiation and counterproductive work behaviors (Bennett and Robinson, 2000; Fox and Spector, 2006). Third, this study contributes to the literature on power distance orientation, which has a negative influence on emotional exhaustion and counterproductive work behaviors, thus extending our growing understanding of the specific conditions of the effect of ingratiation.

## Theory and Hypotheses

### Ingratiation and Counterproductive Work Behaviors

Counterproductive work behaviors refer to voluntary behaviors that violate significant organizational and social norms (Robinson and Bennett, 1995; Spector and Fox, 2005). These behaviors not only cost organization extra billions of dollars annually (Marcus and Schuler, 2004), but also exacerbate the victim’s psychological insecurity (Griffin et al., 1998). However, for employees who engage in counterproductive work behaviors, they tend to consider counterproductive work behaviors as a way to react to perceived workplace stressors and provoked negative affect (Martinko et al., 2002; Spector and Fox, 2005). Cohen-Charash and Mueller (2007) believed that these behaviors are empowering and help compensate for employees’ feeling of inadequacy caused by imbalance in the reciprocal exchange of resources. According to conservation of resources theory, employees might engage in counterproductive work behaviors to acquire information and assistance and to fulfill other needs that can reduce their psychological strain or enable them to obtain work goals (Anderson and Bushman, 2002; Penney et al., 2011). Consistent with this view, Krischer et al. (2010) found that compared with those who refrained from production deviance and withdrawal, employees who engaged in those behaviors encountered less emotional exhaustion.

Ingratiation is a typical impression management tactic and is characterized as a fundamental mechanism by which individuals build and maintain social relationships (Jones, 1964; Kipnis et al., 1980). In the workplace, employees may ingratiate themselves to their supervisors with the goal of having a chance to obtain prestigious appointments or to avoid salary cuts (Kumar and Beyerlein, 1991). Unfortunately, engaging in ingratiation also has a dark side (Bolino et al., 2013); it can backfire and damage employees’ image (Turnley and Bolino, 2001), and it can trigger employees’ unethical behaviors (Keeves et al., 2017). Studies have provided evidence that ingratiation can directly cause employees’ depletion in time, energy and money, which may make employees unable to resist the temptation to perform deviances (Judge et al., 2006; Krischer et al., 2010). For example, Klotz et al. (2018) proposed that the daily use of ingratiation was positively related to employees’ deviance due to the expenditure of self-control resources. Ingratiation may also violate employees’ meritocratic value and threaten their positive self-regard, eliciting subsequent resentment toward their supervisors (Rorty, 2000; Leach and Spears, 2008; Ferro, 2010) and in turn leading to social undermining (Reh et al., 2018).

Conservation of resources theory highlights that individuals strive to obtain, maintain and protect resources that they prize or value, and the potential or real loss of these resources threatens them (Hobfoll, 1989). Engaging in ingratiation may be depleting because it requires effort to minimize the possibility that supervisors perceive that they have ulterior motives (Leary, 1995; Klotz et al., 2018); this consumes self-control resources (Vohs et al., 2005). In this case, employees find that it is difficult to regulate and regain resources, leaving them prone to engaging in counterproductive work behaviors (Bennett and Robinson, 2002; Spector and Fox, 2010). Indeed, studies have shown that employees with low self-control are more likely to engage in certain types of fraud (Yam et al., 2014). Meanwhile, when employees experience the depletion of resources (Homans, 1961; Leventhal, 1976), a perceived imbalance may result, which may motivate them to perform harmful behaviors to restore balance (Masterson et al., 2000; Cropanzano and Mitchell, 2005). Penney et al. (2011) recognized that counterproductive work behaviors were instrumental in reducing the psychological strain linked to resource loss. Thus, we propose the following:

H1: Ingratiation is positively related to counter- productive work behaviors.

### The Mediating Role of Emotional Exhaustion

Emotional exhaustion has been described as a chronic state of emotional and physical fatigue that reflects individuals’ sense of being depleted by their work (Maslach and Jackson, 1981; Maslach et al., 2001). Maslach et al. (2001) pointed out that emotional exhaustion is a psychological syndrome in response to stressors in the workplace, representing the individual stress dimension of burnout (Freudenberger, 1974; Maslach and Jackson, 1981). Emotional exhaustion occurs when employees believe that they no longer have the necessary resources to understand, predict, and control the stressors confronting them (Wright and Hobfoll, 2004; Schaufeli et al., 2009). Scholars have shown that excessive work demands and higher performance goals can stimulate the production of negative affect, putting the body on high alert and resulting in emotional exhaustion (Barling and Macintyre, 1993; Schaufeli and Bakker, 2004). It is reasonable that people who encounter emotional exhaustion tend to be nervous and anxious (Eysenck et al., 2007), with adverse physical reactions such as increased rates of illness (Pines and Maslach, 1978; Cherniss, 1980). In addition to health impacts, other adverse outcomes associated with emotional exhaustion are low job performance, high turnover intention, and avoidant coping behaviors (Tepper et al., 2007; Knudsen et al., 2008; Lv et al., 2012).

According to conservation of resources theory, compared to resource gain, resource loss is more salient and therefore produces stronger cognitive and affective reactions, more specifically, exhaustion (Hobfoll, 1989; Hobfoll, 2001). As described above, engaging in ingratiation can be taxing; it requires employees to envision ways to flatter their supervisor, such as expressing positive emotions and adopting expressive language (Park et al., 2011). Such intentional behaviors require employees to draw attention and cognitive energy from a finite pool of resources (Graen and Scandura, 1987; Graen and Uhl-Bien, 1995), and resource loss is accompanied by threats and pressure (Greenberg, 2004). Additionally, employees may focus on the outcomes after ingratiating, and rumination, anxiety, and depression may tax their mental resources (Eysenck et al., 2007). The failure of ingratiation may exacerbate employees’ negative emotional reaction (Mikula et al., 1998), and dealing with this reaction further depletes their limited resources (Hobfoll, 2001; Sapolsky, 2004). Consequently, personal resources cannot be recovered or continue to be consumed, and employees’ bodies may experience a crash that results in emotional exhaustion (Moss et al., 2003). Bolton et al. (2012) indicated that employees might suffer emotional exhaustion when valued resources are threatened or lost and they were unable to yield anticipated returns.

As a negative affect state, emotional exhaustion may be particularly detrimental for employees’ behaviors in the workplace (Sonnentag and Frese, 2003). Whitman et al. (2014) noted that to mitigate stress and conserve resources, emotionally exhausted employees tended to engage in feedback avoidance toward their supervisor. Welsh et al. (2020) pointed out that employees who are experiencing emotional exhaustion reduce their engagement in citizenship behavior due to insufficient resources. Emotional exhaustion can decrease self-control (Baumeister et al., 1998) and impel employees to take unauthorized breaks and harm others (Jones, 1980, 1981). Consistent with conservation of resources theory, individuals with scarce resources are inclined to enhance and buttress their resources against further damage and loss by engaging in withdrawal and destructive behaviors (Hobfoll et al., 1990; Halbesleben, 2006). Employees who suffer emotional exhaustion have difficulty regulating their negative emotional states (Mikula et al., 1998) and thus entertain thoughts of engaging in counterproductive work behaviors, which serve as an affect-regulation technique (Baumeister et al., 1996; Bushman et al., 2001). Indeed, counterproductive work behaviors can create an emotional buffer between employees and demanding situations (Taris et al., 2001), which may help them maintain their resources and reduce threat. There is evidence that emotional exhaustion is a direct predictor of counterproductive work behaviors (Watson et al., 1988; Bolton et al., 2012). Therefore, we propose the following:

H2: Emotional exhaustion mediates the positive relationship between ingratiation and counter- productive work behaviors.

### The Moderating Role of Power Distance Orientation

As one of Hofstede’s (1980) four cultural value dimensions, power distance has garnered significant interest from numerous scholars (Farh et al., 2007; Lin et al., 2013; Meydan et al., 2014). Although Hofstede (1980) argued that cultural values are meaningful at the societal level, researchers have found that each of his value dimensions has large variation over individuals in societies and that these individual differences have direct effects on many outcomes (Clugston et al., 2000; Kirkman and Shapiro, 2001). In the current study, we focus on the individual power distance orientation that reflects the degree to which individuals differ in their perceptions of unequal power distribution in organizations (Dorfman and Howell, 1988; Kirkman et al., 2009). Previous studies have suggested that organizational culture, leadership style, and heterogeneity between supervisors and subordinates are important antecedents of power distance orientation (Hofstede, 2001; Roberge and Van Dick, 2010). Employees with high power distance orientation typically obey instruction without question and accept top-down and one-way direction from their supervisor (Javidan et al., 2006). These employees may believe that it is reasonable to flatter and show opinion conformity to their supervisor. In contrast, employees who have low power distance orientation view that their interaction with their supervisor is equal and that employees and supervisors differ only in terms of their working power and responsibility (Chen and Aryee, 2007). Such employees may not ingratiate. In other words, employees with different power distance orientations may have different perceptions of ingratiation and thus different response to these behaviors (Lin et al., 2013).

Conservation of resources theory holds that certain personality traits influence reactions to the process of gaining resources and avoiding resource loss (Hobfoll et al., 1990; Koopman et al., 2016). This means that the way individuals interpret environmental stimuli can influence their evaluation of resources and their response to stressors (Folkman and Lazarus, 1984). High power distance orientation makes employees receptive to more role-constrained interaction with their supervisor (Auh et al., 2016) and to the imbalance of power (Tyler et al., 2000). They take opinion conformity, flattery, and other enhancement for granted and perceive that the resources to engage in these behaviors are less likely to be drained, which results in less emotional exhaustion (Maslach et al., 2001). Further, employees with high power distance orientation may not worry about the consequence of ingratiation, which requires less consumption of emotional resources for these employees than for employees with low power distance orientation (Mikula et al., 1998; Lian et al., 2012). In contrast, employees with low power distance orientation perceive that conflict with and criticism of authority figures are appropriate (Tyler et al., 2000; Farh et al., 2007) and treat supervisors as similar others (Loi et al., 2012). These employees are more sensitive to resource loss caused by ingratiation and encounter more mental stress; thus, they more easily experience emotional exhaustion. As such, we hypothesize the following:

H3: Power distance orientation moderates the positive relationship between ingratiation and emotional exhaustion, such that the relationship is stronger (vs. weaker) when employees have lower (vs. higher) power distance orientation.

In addition, we argue that power distance orientation may play a moderating role in the indirect effect of emotional exhaustion on the relationship between ingratiation and counterproductive work behaviors. According to conservation of resources theory, people who lack resources are vulnerable to suffering more resource loss, and subsequent resource gains can help offset the effect of this loss (Hobfoll, 1989; Lin et al., 2019). Ingratiation directly consumes employees’ resources (Graen and Uhl-Bien, 1995; Klotz et al., 2018), and they can either induce strain or lead to depression, thus creating stronger feelings of emotional exhaustion (Maslach et al., 2001; Lin et al., 2019). To maintain resources and recover lost resources, employees may resort to counterproductive work behaviors as a form of compensation (Penney et al., 2011). Employees with high power distance orientation view ingratiation as a way to gain resources and are less sensitive to the depletion of resources; some negative affect inherent to emotional exhaustion may disappear (Sapolsky, 2004; Eysenck et al., 2007). Furthermore, employees believe that the positive outcomes of ingratiation are sufficient to offset the loss of resources (Hobfoll, 2001), resulting in less counterproductive work behavior. Conversely, employees who have low power distance orientation may perceive threats and stress from the loss of resources caused by ingratiation, and they are unable to deal with the subsequent negative affect, which leads to more emotional exhaustion and in turn leads to more counterproductive work behaviors. Therefore, we propose the following:

H4: Power distance orientation moderates the mediating effect of emotional exhaustion on the relationship between ingratiation and counterproductive work behaviors, such that the mediating effect of emotional exhaustion is stronger (vs. weaker) among employees with lower (vs. higher) power distance orientation.

## Materials and Methods

### Participants and Procedures

Time-lagged data were collected from supervisor-employee dyads at six manufacturing firms in China. In order to ensure the smoothest progress of the survey, we first contacted the managers who have the authority to take charge of the questionnaire survey, and then introduced our academic purpose and highlighted the anonymity in our survey to the participants. The data were collected at 3 time points. At Time 1, the employees reported their ingratiation behavior and power distance orientation. After one month (Time 2), the same employees reported their emotional exhaustion. Another month later (Time 3), the supervisors rated the employees’ counterproductive work behaviors.

We used identified survey method to give each participant a unique code to make sure that we could gather matched data. For example, if the supervisor is numbered A, the three subordinates are A1, A2, and A3 respectively. At Time 1, 395 participants took part in the first data collection session. At Time 2, we recovered 302 valid questionnaires. At Time 3, we issued questionnaires to the supervisors according to the numbered roster. Finally, 61 supervisors and 216 employees completed the survey, for 216 matched supervisor-employee dyads. The valid response rate was 54.7%. Supervisors were an average of 39 years old, and 63.9% were male. A total of 62.3% had a bachelor’s degree, and their job tenure was longer than 3 years. Employees were an average of 28 years, 64.4% were female. Regarding education, 33.8% had a high school degree, 56.6% had a bachelor’s degree, and 9.7% had a master’s degree or higher. Approximately 86.1% of employees’ organizational tenure was longer than one year.

### Measures

All scales used in the survey were well established by previous studies. We followed a translation and back-translation procedure to ensure the accuracy of the scales (Brislin, 1986). All items used a Likert-type scale ranging from 1 (strongly disagree) to 5 (strongly agree).

#### Ingratiation

Employees reported their ingratiatory behavior using a 4-item scale developed by Bolino and Turnley (1999). A sample item is “I praised my supervisor for his accomplishments.” Cronbach’s alpha for this scale was 0.88.

#### Emotional Exhaustion

Employees reported their emotional exhaustion using a 5-item scale developed by Schaufeli et al. (1996). A sample item is “I feel emotionally drained from my work.” Cronbach’s alpha for this scale was 0.94.

#### Power Distance Orientation

Employees reported their power distance orientation using an 8-item scale developed by Kirkman et al. (2009). A sample item is “Supervisors should be able to make the right decisions without consulting others.” Cronbach’s alpha for this scale was 0.71.

#### Counterproductive Work Behaviors

Supervisors were asked to rate employees’ counterproductive work behaviors using a 19-item scale developed by Bennett and Robinson (2000) that contained items related to organizational counterproductive work behaviors (13 items) and interpersonal counterproductive work behaviors (6 items). We noted that one item, “Made an ethnic, religious, or racial remark at work,” was unsuitable in the Chinese context, so we removed it from the formal survey. The Cronbach’s alpha of organizational and interpersonal counterproductive work behaviors was 0.94 and 0.95, respectively. The overall Cronbach’s alpha of counterproductive work behaviors was 0.96.

We controlled for the possible effects of employees’ gender (0 = male, 1 = female), age (years), education level (1 = senior middle school or less, 2 = college or associate’s degree, 3 = bachelor’s degree, 4 = master’s degree or above), and job tenure (years), since studies have suggested that these factors might affect employees’ counterproductive work behaviors (Lau et al., 2003).

### Analytic Strategy

We first conducted confirmatory factor analysis to examine the discriminant validity of all variables. And then we employed regression analysis to preliminary examine the relationship proposed in our theoretical model. In addition, we used bootstrap resampling with Mplus to test the mediating effect of emotional exhaustion and the moderating effect of power distance orientation. Finally, we adopted the moderation path analysis introduced by Edwards and Lambert (2007) and used bootstrap resampling to test for indirect effects at one standard deviation above the mean and one standard deviation below the mean of the moderator.

## Results

### Confirmatory Factor Analysis

Confirmatory factor analysis was conducted to test the discriminant validity of all variables. We set up a model with four factors: ingratiation, emotional exhaustion, power distance orientation and counterproductive work behaviors. The results in Table 1 indicate that the fitting effect of the four-factor model (χ^2^/*df* = 1.94, TLI = 0.91, CFI = 0.92, RMSEA = 0.07) was much better than that of the other models. The results suggested that the fitting index supported the four-factor model, which meant that our four constructs had good discriminant validity.

**TABLE 1:** Results of confirmatory factor analysis.

Model	Factor structure	χ^2^/*df*	TLI	CFI	RMSEA
Four-factor Model	Ingratiation; Emotional exhaustion; Power distance orientation; Counterproductive work behaviors	1.94	0.91	0.92	0.07
Three-factor Model	Ingratiation and Emotional exhaustion were combined into one factor	3.98	0.70	0.722	0.12
Two-factor Model	Ingratiation, Emotional exhaustion and Power distance orientation were combined into one factor	4.79	0.62	0.64	0.13
One-factor Model	All factors combined into one factor	6.27	0.46	0.50	0.16

### Descriptive Analysis

The means, standard deviations, and correlations of the variables are provided in Table 2. The results showed that counterproductive work behaviors were significantly related to ingratiation (*r* = 0.22, *p* < 0.01) and emotional exhaustion (*r* = 0.29, *p* < 0.01). Emotional exhaustion was significantly related to ingratiation (*r* = 0.15, *p* < 0.05), which was consistent with our hypotheses.

**TABLE 2:** Means, standard deviations, and correlations.

Variables	1	2	3	4	5	6	7	8
Mean	1.64	28.36	2.73	6.45	2.78	2.24	2.73	1.94
SD	0.48	6.04	0.68	5.97	0.72	0.79	0.54	0.67
(1) Gender								
(2) Age	−0.07	–						
(3) Education	−0.13	0.14*	–					
(4) Tenure	−0.05	0.96**	−0.12	–				
(5) Ingratiation	−0.01	−0.01	0.01	−0.01	–			
(6) Emotional exhaustion	0.02	0.13*	0.04	0.10	0.15*	–		
(7) Power distance orientation	−0.04	0.15*	0.11	0.12	0.08	−0.03	–	
(8) Counterproductive work behaviors	−0.09	0.01	0.00	−0.01	0.22**	0.29**	−0.02	–

*p < 0.05, **p < 0.01.

### Hypotheses Testing

We used the Mplus 7.0 to test the hypotheses. We performed regression analyses between the ingratiation and control variables and counterproductive work behaviors in Table 3. Model 6 revealed that ingratiation was positively related to counterproductive work behaviors (β = 0.23, *p* < 0.01). Therefore, H1 was supported.

**TABLE 3:** Regression analyses for hypothesis testing.

Predictor	Emotional exhaustion				Counterproductive work behaviors			
		
	M1	M2	M3	M4		M5	M6	M7
Gender	0.02	0.02	0.02	0.04		−0.10	−0.10	−0.10
Age	1.21	1.24*	1.26	1.34		0.65	0.69	0.36
Education	−0.25	−0.26*	−0.26	−0.27		−0.18	−0.19	−0.13
Tenure	−1.09	−0.11*	−1.13	−0.20		0.66	−0.69	0.40
Ingratiation		0.15*	0.16*	0.14*			0.23**	0.19**
Emotional exhaustion								0.26**
Power distance orientation			−0.07	−0.05				
Ingratiation × Power distance orientation				−0.17*				
*R*^2^	0.04	0.07*	0.07	0.10*		0.02	0.07	0.13

*p < 0.05, **p < 0.01.

We used bootstrap resampling (5000 times) to test the mediation model following the recommendations of Preacher and Hayes (2008). As the path coefficient results in Table 4 show, the coefficient for the indirect effect of emotional exhaustion was 0.10, and the bias-corrected 95% confidence interval excluded zero (95% CI [0.01, 0.18]), thus supporting H2.

**TABLE 4:** Results of mediation effect analysis.

Variables	Ingratiation → Emotional exhaustion →Counterproductive work behaviors	
	
	Effect	95% Confidence interval
Gender	−0.14(0.10)	[−0.32, 0.16]
Age	0.08(0.06)	[−0.03, 0.19]
Education	−0.19(0.14)	[−0.48, 0.07]
Tenure	−0.08(0.06)	[−0.19, 0.04]
Indirect Effect	0.10(0.04)	[0.01, 0.18]
Direct Effect	0.21(0.07)	[0.06, 0.35]
Total Effect	0.31(0.09)	[0.12, 0.49]

As model 4 in Table 3 shows, the interaction between ingratiation and power distance orientation was negatively related to emotional exhaustion (β = −0.17, *p* < 0.05); thus, H3 was supported. To further examine the moderation model, we used bootstrap resampling (5000 times). The results showed that power distance orientation negatively moderated the relationship between ingratiation and emotional exhaustion (95% CI [−0.50, −0.11]). The simple effects analyses in Table 5 suggested that when power distance orientation was high, the coefficient was not significant (*b* = −0.01, CI [−0.22, 0.21]). When power distance orientation was low, however, the coefficient was significant (*b* = 0.32, 95% CI [0.16, 0.49]). The difference between high and low power distance orientations was also significant (*b* = −0.33, 95% CI [−0.59, −0.08]).

**TABLE 5:** Results of moderation effect analysis.

Variables	Ingratiation → Emotional exhaustion	
	
	Coefficient	95% Confidence interval
Gender	0.06(0.11)	[−0.17, 0.26]
Age	0.18 (0.06)	[0.06, 0.29]
Education	−0.31(0.17)	[−0.61, −0.02]
Tenure	−0.16 (0.06)	[−0.28, −0.04]
High Power distance orientation	−0.01(0.11)	[−0.22, 0.21]
Low Power distance orientation	0.32(0.08)	[0.16, 0.49]
Differences	−0.33(0.13)	[−0.59, −0.08]
		

**FIGURE 1:**
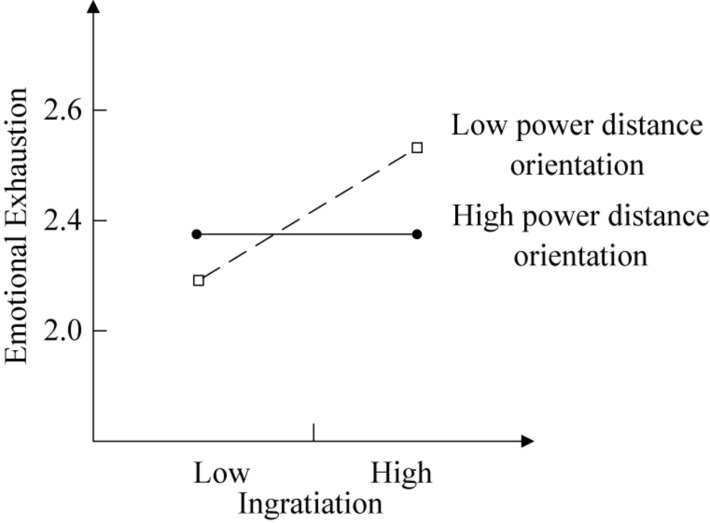
Moderating effect of power distance orientation on the relationship between ingratiation and emotional exhaustion.

To better interpret the interaction patterns, we draw Figure 1. As Figure 1 shows that when power distance orientation was high, emotional exhaustion was not significantly influenced by ingratiation, indicating that H3 was supported.

For H4, we adopted the moderation path analysis introduced by Edwards and Lambert (2007) and used bootstrap resampling (5000 times) to test for indirect effects at one standard deviation above the mean and one standard deviation below the mean of the moderator. The results in Table 6 show that the direct effect was not significant when power distance orientation was high (*b* = 0.00, 95% CI [−0.05, 0.05]. However, the indirect effect was significant when power distance orientation was low (*b* = 0.07, 95% CI [0.03, 0.14]). The difference between high and low power distance orientations was also significant (*b* = −0.07, 95% CI [−0.16, −0.02]). Therefore, H4 was supported.

**TABLE 6:** Results of moderated moderation effect analysis.

Variables	Ingratiation → Emotional exhaustion →Counterproductive work behaviors	
	
	Coefficient	95% Confidence interval
Gender	−0.14 (0.10)	[−0.33, 0.05]
Age	0.04 (0.06)	[−0.07, 0.14]
Education	−0.13(0.13)	[−0.40, −0.13]
Tenure	−0.05(0.06)	[−0.15, 0.07]
High Power distance orientation	0.00(0.03)	[−0.05, 0.05]
Low Power distance orientation	0.07(0.03)	[0.03, 0.14]
Differences	−0.07(0.04)	[−0.16, −0.02]

## Discussion

In many organizations, ingratiation has become one of the most important influence tactics used by employees toward their supervisor due to the imbalance of power and status between them. As a result, considerable research attention has been devoted to its detrimental outcomes (Westphal and Stern, 2006; Keeves et al., 2017; Klotz et al., 2018). Drawing from conservation of resources theory, we theorized and tested how ingratiation links to counterproductive work behaviors. The results show that ingratiation had a positive effect on counterproductive work behaviors, and emotional exhaustion played a mediating role in this positive relationship. Meanwhile, power distance orientation negatively moderated the relationship between ingratiation and emotional exhaustion and the indirect effect of emotional exhaustion on the relationship between ingratiation and counterproductive work behaviors. Specifically, the conditional indirect effect of ingratiation on counterproductive work behaviors through emotional exhaustion was more positive when power distance orientation was low than when it was high.

### Theoretical Contributions

First, this study contributes to the literature on ingratiation by highlighting its predictive effect on counterproductive work behaviors based on conservation of resources theory. Previous studies have focused mainly on the positive outcomes of ingratiation (Higgins et al., 2003; Cooper, 2005; Westphal and Stern, 2006), while little theory or research has addressed its downsides (Crant, 1996; Turnley and Bolino, 2001). In particular, it is not well understood whether ingratiation has an effect on the ingratiator’s work behaviors. As such, this study fills this gap in the literature by revealing that employees’ ingratiatory behaviors have a positive effect on their subsequent counterproductive work behaviors. Ingratiation often involves adopting not only expressive language but also non-verbal expressions (Park et al., 2011; Klotz et al., 2018). Such behaviors require employees to expend their valued personal resources, resulting in resource depletion (Lanaj et al., 2016). According to conservation of resources theory, employees strive to engage in counterproductive work behaviors to maintain their resources and to prevent further damage and loss to these resources (Wright and Cropanzano, 1998; Hobfoll, 2002). This study offers proof of the deleterious effects related to ingratiation in the workplace and provides a new perspective to explain why ingratiation sometimes backfires.

Second, this study helps open the “black box” and extends the understanding of how ingratiation influences counterproductive work behaviors by identifying emotional exhaustion as a mediator from the perspective of conservation of resources theory. Ingratiation can be seen as emotional labor for employees, as it requires the consumption of cognitive energy and mental resources in the preparation for and execution of ingratiation (Graen and Uhl-Bien, 1995; Eysenck et al., 2007), which leads to emotional exhaustion. This is consistent with conservation of resources theory, which holds that individuals are more likely to encounter emotional exhaustion when experiencing resource threat and loss (Hobfoll, 1989, 2001). As a negative affective state, emotional exhaustion may result in a decrease in self-control (Baumeister et al., 1998) and then impel employees to perform counterproductive work behaviors (Robinson and Bennett, 1995; Spector and Fox, 2005), which help employees contribute to their resource reservoir and compensate for resource loss. Examining the mediating effect of emotional exhaustion through the lens of conservation of resources theory reveals a rich theoretical mechanism of the relationship between ingratiation and counterproductive work behaviors. To a certain extent, this study will be a valuable supplement to the theory.

Third, this study reveals a specific condition of ingratiation effects, that is, the moderating role of power distance orientation. Although ingratiation is prevalent in many organizations, it is particularly significant in the Chinese context (Aryee and Debrah, 1993). As such, this study examined the construct of power distance orientation in the Chinese cultural context, which might have an effect on employees’ perception of ingratiation. Previous studies have mainly explored the undesirable outcomes of power distance orientation (Carl et al., 2004; Farh et al., 2007; Kirkman et al., 2009). However, employees with high power distance orientation typically obey instruction without question and accept top-down direction from their supervisor (Javidan et al., 2006). These employees may take ingratiation for granted and be less sensitive to the loss of resources (Mikula et al., 1998); thus, they may have a lower likelihood of experiencing emotional exhaustion and engaging in counterproductive behavior. The results of this study extend our understanding of how the effect of ingratiation manifests at work and enrich the literature on power distance orientation by providing evidence of its positive effects.

### Practical Implications

In addition to their theoretical contributions, our findings provide guidance for managerial practices. First, managers should pay attention to the detrimental outcomes of ingratiation. The results of this study suggest that ingratiation is depleting (Lanaj et al., 2016) and thus has a positive effect on emotional exhaustion and counterproductive work behaviors that can damage organizational performance. As such, managers should strive to create an open, transparent, authentic, mutual trust, and information sharing working environment, and encourage employees to act in a way that is consistent with one’s true self (Ilies et al., 2005). Meanwhile, managers can advocate the ideal of authenticity in communications with others and the belief of working hard (Keeves et al., 2017; Thorisdottir et al., 2009). In addition, managers should value not the behavioral factors that exceed normal work behaviors but the personal ability of employees when making decision and balance the relationship with employees, which will promote them to reduce the engagement in ingratiation.

Second, organizations should be alerted to the double-edged effects of power distance orientation. One of the primary findings of this study is that power distance orientation can mitigate the negative consequences of ingratiation. On the one hand, employees with a high power distance orientation regard ingratiation as an intra-role behavior and thus are unable to perceive a reduction in their personal resources (Mikula et al., 1998); thus, ingratiation results in less harmful outcomes. On the other hand, high power orientation may make employees perceive less disagreement and conflict with management in organizations (Chen and Aryee, 2007; Kirkman et al., 2009); thus, it is possible for them to ingratiate themselves to their supervisor. To minimize ingratiation, organizations should attach importance to creating an equal and open working environment, a free and democratic corporate culture or a flattened organizational structure.

### Limitations and Directions for Future Research

This study has some limitations that suggest fruitful directions for future research. First, this study examined the effect of ingratiation on the ingratiator’s behavior, but we did not consider whether the outcomes of ingratiation might influence this relationship. Future research can use a lagged design to explore the effect of the success or failure of ingratiation on work behaviors. Second, this study adopted a sub-scale of impression management scale developed by foreign scholars, which may not be applicable to Chinese contexts. Future research can attempt to develop scales of ingratiatory behavior in the Chinese cultural context. Third, the current study was conducted in the Chinese context; thus, there is no certainty that our results would remain valid in Western culture. Future research can design cross-cultural research to test the generalizability of the results of this study. Finally, the limited sample size is a limitation in our research, which may lead to biased estimation results. Future research can expand the sample size to test the results.

## Data Availability Statement

The raw data supporting the conclusions of this article will be made available by the authors, without undue reservation.

## Ethics Statement

Ethical review and approval was not required for the study on human participants in accordance with the local legislation and institutional requirements. Written informed consent from the participants was not required to participate in this study in accordance with the national legislation and the institutional requirements.

## Author Contributions

All authors listed have made a substantial, direct and intellectual contribution to the work, and approved it for publication.

## Conflict of Interest

The authors declare that the research was conducted in the absence of any commercial or financial relationships that could be construed as a potential conflict of interest.

## References

[B1] Anderson C. A. Bushman B. J. (2002). Human aggression. *Annu. Rev. Psychol.* 53 27–51. 11752478 10.1146/annurev.psych.53.100901.135231

[B2] Aryee S. Debrah Y. A. (1993). A cross-cultural application of a career planning model. *J. Organ. Behav.* 14 119–127. 10.1002/job.4030140203

[B3] Aryee S. Wyatt T. Stone R. (1996). Early career outcomes of graduate employees: the effect of mentoring and ingratiation. *J. Manag. Stud.* 33 95–118. 10.1111/j.1467-6486.1996.tb00800.x

[B4] Auh S. Menguc B. Spyropoulou S. Wang F. (2016). Service employee burnout and engagement: the moderating role of power distance orientation. *J. Acad. Mark. Sci.* 44 726–745. 10.1007/s11747-015-0463-4

[B5] Barling J. Macintyre A. T. (1993). Daily work role stressors, mood and emotional exhaustion. *Work Stress* 7 315–325. 10.1080/02678379308257071

[B6] Baumeister R. F. Bratslavsky E. Muraven M. Tice D. M. (1998). Ego depletion: is the active self a limited resource? *J. Pers. Soc. Psychol.* 74 1252–1265. 10.1037/0022-3514.74.5.1252 9599441

[B7] Baumeister R. F. Smart L. Boden J. M. (1996). Relation of threatened egotism to violence and aggression: the dark side of high self-esteem. *Psychol. Rev.* 103 5–33. 10.1037/0033-295x.103.1.5 8650299

[B8] Bennett R. J. Robinson S. L. (2000). Development of a measure of workplace deviance. *J. Appl. Psychol.* 85 349–360. 10.1037/0021-9010.85.3.349 10900810

[B9] Bennett R. J. Robinson S. L. (2002). “The Past, Present and Future of Workplace Deviance Research,” in *Organizational Behavior: The State of the Science*, 2nd Edn, ed. Greenberg J. (Mahwah, NJ: Erlbaum), 247–281.

[B10] Bolino M. C. Klotz A. C. Turnley W. H. Harvey J. (2013). Exploring the dark side of organizational citizenship behavior. *J. Organ. Behav.* 34 542–559. 10.1002/job.1847

[B11] Bolino M. C. Turnley W. H. (1999). Measuring impression management in organizations: a scale development based on the jones and pittman taxonomy. *Organ. Res. Methods* 2 187–206. 10.1177/109442819922005

[B12] Bolton L. R. Harvey R. D. Grawitch M. J. Barber L. K. (2012). Counterproductive work behaviors in response to emotional exhaustion: a moderated mediational approach. *Stress Health* 28 222–233. 10.1002/smi.1425 22281803

[B13] Brislin R. (1986). “The wording and translation of research instruments,” in *Field Methods in Cross-cultural Research*, eds Lonner W. J. Berry J. W. (Beverly Hills, CA: Sage), 137–164.

[B14] Bushman B. J. Baumeister R. F. Phillips C. M. (2001). Do people aggress to improve their mood? Catharsis beliefs, affect regulation opportunity, and aggressive responding. *J. Pers. Soc. Psychol.* 81 17–32. 10.1037/0022-3514.81.1.17 11474722

[B15] Carl D. Gupta V. Javidan M. (2004). “Power distance,” in *Culture, Leadership and Organizations: The GLOBE Study of 62 Societies*, eds House R. J. Hanges P. J. Javidan M. Dorfman P. W. Gupta V. (Thousand Oaks, CA: Sage).

[B16] Chen Z. X. Aryee S. (2007). Delegation and employee work outcomes: an examination of the cultural context of mediating processes in China. *Acad. Manag. J.* 50 226–238. 10.5465/amj.2007.24162389

[B17] Cherniss C. (1980). *Professional Burnout in Human Service Organizations.* Westport: Praeger Publishers.

[B18] Clugston M. Howell J. P. Dorfman P. W. (2000). Does cultural socialization predict multiple bases and foci of commitment? *J. Manag.* 26 5–30. 10.1177/014920630002600106

[B19] Cohen-Charash Y. Mueller J. S. (2007). Does perceived unfairness exacerbate or mitigate interpersonal counterproductive work behaviors related to envy? *J. Appl. Psychol.* 92 666–680. 10.1037/0021-9010.92.3.666 17484549

[B20] Cooper C. D. (2005). Just joking around? Employee humor expression as an ingratiatory behavior. *Acad. Manag. Rev.* 30 765–776. 10.5465/amr.2005.18378877

[B21] Crant J. M. (1996). Doing more harm than good: when is impression management likely to evoke a negative response? *J. Appl. Soc. Psychol.* 26 1454–1471. 10.1111/j.1559-1816.1996.tb00080.x

[B22] Cropanzano R. Mitchell M. S. (2005). Social exchange theory: an interdisciplinary review. *J. Manag.* 31 874–900. 10.1177/0149206305279602

[B23] Dorfman P. W. Howell J. P. (1988). Dimensions of national culture and effective leadership patterns: hofstede revisited. *Adv. Int. Comp. Manag.* 3 127–150.

[B24] Edwards J. R. Lambert L. S. (2007). Methods for integrating moderation and mediation: a general analytical framework using moderated path analysis. *Psychol. Methods* 12 1–22. 10.1037/1082-989x.12.1.1 17402809

[B25] Eysenck M. W. Derakshan N. Santos R. Calvo M. G. (2007). Anxiety and cognitive performance: attentional control theory. *Emotion* 7 336–353. 10.1037/1528-3542.7.2.336 17516812

[B26] Farh J. L. Hackett R. D. Liang J. (2007). Individual-level cultural values as moderators of perceived organizational support–employee outcome relationships in china: comparing the effects of power distance and traditionality. *Acad. Manag. J.* 50 715–729. 10.5465/amj.2007.25530866

[B27] Ferro M. (2010). *Resentment in History.* Malden, MA: Polity.

[B28] Folkman S. Lazarus R. S. (1984). *Stress, Appraisal, and Coping.* New York, NY: Springer.

[B29] Fox S. Spector P. E. (2006). The many roles of control in a stressor-emotion theory of counterproductive work behavior. *Res. Occup. Stress Well Being* 5 171–201. 10.1016/s1479-3555(05)05005-5

[B30] Freudenberger H. J. (1974). Staff-burnout. *J. Soc. Issues* 30 159–165.

[B31] Gino F. Schweitzer M. E. Mead N. L. Ariely D. (2011). Unable to resist temptation: how self-control depletion promotes unethical behavior. *Organ. Behav. Hum. Dec. Process.* 115 191–203. 10.1016/j.obhdp.2011.03.001

[B32] Graen G. B. Scandura T. A. (1987). Toward a psychology of dyadic organizing. *Res. Organ. Behav.* 9 175–208.

[B33] Graen G. B. Uhl-Bien M. (1995). Development of leader-member exchange (LMX) theory of leadership over 25 years: applying a multi-level multi-domain perspective. *Leadersh. Q.* 6 219–247. 10.1016/1048-9843(95)90036-5

[B34] Grant R. M. (1996). Toward a knowledge-based theory of the firm. *Strateg. Manag. J.* 17 109–122. 10.1002/smj.4250171110

[B35] Greenberg L. S. (2004). Emotion–focused therapy. *Clin. Psychol. Psychother.* 11 3–16. 10.1002/cpp.624 19639649

[B36] Griffin R. W. O’Leary-Kelly A. Collins J. (1998). “Dysfunctional work behaviors in organizations,” in *Trends in organizational behavior*, Vol. 5 eds Cooper C. L. Rousseau D. M. (New York: Wiley), 65–82.

[B37] Guglielmi D. Mazzetti G. Villano P. Topa Cantisano G. (2018). The impact of perceived effort–reward imbalance on workplace bullying: also a matter of organizational identification. *Psychol. Health Med.* 23 511–516. 10.1080/13548506.2017.1363396 28792231

[B38] Halbesleben J. R. (2006). Sources of social support and burnout: a meta-analytic test of the conservation of resources model. *J. Appl. Psychol.* 91 1134–1145. 10.1037/0021-9010.91.5.1134 16953774

[B39] Higgins C. A. Judge T. A. (2004). The effect of applicant influence tactics on recruiter perceptions of fit and hiring recommendations: a field study. *J. Appl. Psychol.* 89 622–632. 10.1037/0021-9010.89.4.622 15327349

[B40] Higgins C. A. Judge T. A. Ferris G. R. (2003). Influence tactics and work outcomes: a meta-analysis. *J. Organ. Behav.* 24 89–106. 10.1002/job.181

[B41] Hobfoll E. (1989). Conservation of resources: a new attempt at conceptualizing stress. *Am. Psychol.* 44 513–524. 10.1037/0003-066x.44.3.513 2648906

[B42] Hobfoll S. E. (2001). The influence of culture, community, and the nested-self in the stress process: advancing conservation of resources theory. *Appl. Psychol.* 50 337–421. 10.1111/1464-0597.00062

[B43] Hobfoll S. E. (2002). Social and psychological resources and adaptation. *Rev. Gen. Psychol.* 6 307–324. 10.1037/1089-2680.6.4.307

[B44] Hobfoll S. E. Freedy J. Lane C. Geller P. (1990). Conservation of social resources: social support resource theory. *J. Soc. Pers. Relationsh.* 7 465–478. 10.1177/0265407590074004

[B45] Hofstede G. (1980). Motivation, leadership, and organization: do american theories apply abroad? *Organ. Dyn.* 9 42–63. 10.1016/0090-2616(80)90013-3

[B46] Hofstede G. (2001). *Culture’s Consequences: Comparing Values, Behaviors, Institutions and Organizations Across Nations.* Thousand Oaks, CA: Sage publications.

[B47] Homans G. C. (1961). *Social Behavior: Its Elementary Forms.* Brace: Harcourt, 488–531.

[B48] Ilies R. Morgeson F. P. Nahrgang J. D. (2005). Authentic leadership and eudaemonic well-being: understanding leader–follower outcomes. *Leadersh. Q.* 16 373–394. 10.1016/j.leaqua.2005.03.002

[B49] Javidan M. House R. J. Dorfman P. W. Hanges P. J. De Luque M. S. (2006). Conceptualizing and measuring cultures and their consequences: a comparative review of GLOBE’s and Hofstede’s approaches. *J. Int. Bus. Stud.* 37 897–914. 10.1057/palgrave.jibs.8400234

[B50] Johnson D. E. Erez A. Kiker D. S. Motowidlo S. J. (2002). Liking and attributions of motives as mediators of the relationships between individuals’ reputations, helpful behaviors and raters’ reward decisions. *J. Appl. Psychol.* 87 808–815. 10.1037/0021-9010.87.4.808 12184583

[B51] Jones E. E. (1964). *Ingratiation, Social Psychological Analysis.* New York, NY: Meredith Publishing.

[B52] Jones J. W. (1981). *Staff Burnout and Employee Counterproductivity. The Burnout Syndrome: Current Research, Theory, and Interventions.* New York, NY: London House Press.

[B53] Jones T. M. (1980). Corporate social responsibility revisited, redefined. *Calif. Manag. Rev.* 22 59–67. 10.2307/41164877

[B54] Judge T. A. Scott B. A. Ilies R. (2006). Hostility, job attitudes, and workplace deviance: test of a multilevel model. *J. Appl. Psychol.* 91 126–138. 10.1037/0021-9010.91.1.126 16435943

[B55] Keeves G. D. Westphal J. D. McDonald M. L. (2017). Those closest wield the sharpest knife: how ingratiation leads to resentment and social undermining of the CEO. *Admin. Sci. Q.* 62 484–523. 10.1177/0001839216686053

[B56] Kipnis D. Schmidt S. M. Wilkinson I. (1980). Intraorganizational influence tactics: explorations in getting one’s way. *J. Appl. Psychol.* 65 440–452. 10.1037/0021-9010.65.4.440

[B57] Kirkman B. L. Chen G. Farh J. L. Chen Z. X. Lowe K. B. (2009). Individual power distance orientation and follower reactions to transformational leaders: a cross-level, cross-cultural examination. *Acad. Manag. J.* 52 744–764. 10.5465/amj.2009.43669971

[B58] Kirkman B. L. Shapiro D. L. (2001). The impact of cultural values on job satisfaction and organizational commitment in self-managing work teams: the mediating role of employee resistance. *Acad. Manag. J.* 44 557–569. 10.5465/3069370 3069370

[B59] Klotz A. C. He W. Yam K. C. Bolino M. C. Wei W. Houston L. III (2018). Good actors but bad apples: deviant consequences of daily impression management at work. *J. Appl. Psychol.* 103 1145–1154. 10.1037/apl0000335 29939036

[B60] Knudsen H. K. Ducharme L. J. Roman P. M. (2008). Clinical supervision, emotional exhaustion, and turnover intention: a study of substance abuse treatment counselors in the clinical trials network of the national institute on drug abuse. *J. Subst. Abuse Treat.* 35 387–395. 10.1016/j.jsat.2008.02.003 18424048 PMC2637454

[B61] Koopman J. Lanaj K. Scott B. A. (2016). Integrating the bright and dark sides of OCB: a daily investigation of the benefits and costs of helping others. *Acad. Manag. J.* 59 414–435. 10.5465/amj.2014.0262

[B62] Krischer M. M. Penney L. M. Hunter E. M. (2010). Can counterproductive work behaviors be productive? CWB as emotion-focused coping. *J. Occup. Health Psychol.* 15 154–166. 10.1037/a0018349 20364913

[B63] Kumar K. Beyerlein M. (1991). Construction and validation of an instrument for measuring ingratiatory behaviors in organizational settings. *J. Appl. Psychol.* 76 619–627. 10.1037/0021-9010.76.5.619

[B64] Lam W. Huang X. Snape E. D. (2007). Feedback-seeking behavior and leader-member exchange: do supervisor-attributed motives matter? *Acad. Manag. J.* 50 348–363. 10.5465/amj.2007.24634440

[B65] Lanaj K. Johnson R. E. Wang M. (2016). When lending a hand depletes the will: the daily costs and benefits of helping. *J. Appl. Psychol.* 101 1097–1110. 10.1037/apl0000118 27149605

[B66] Lau V. C. Au W. T. Ho J. M. (2003). A qualitative and quantitative review of antecedents of counterproductive behavior in organizations. *J. Bus. Psychol.* 18 73–99.

[B67] Leach C. W. Spears R. (2008). “A vengefulness of the impotent”: the pain of in-group inferiority and schadenfreude toward successful out-groups. *J. Pers. Soc. Psychol.* 95 1383–1396. 10.1037/a0012629 19025290

[B68] Leary M. R. (1995). *Self-Presentation: Impression Management and Interpersonal Behavior.* Boulder, CO: Westview Press.

[B69] Leiter M. P. (1993). “Burnout as a developmental process: consideration of models,” in *Series in Applied Psychology: Social Issues and Questions. Professional Burnout: Recent Developments in Theory and Research*, eds Schaufeli W. B. Maslach C. Marek T. (Boca Raton, FL: Taylor & Francis).

[B70] Leventhal G. S. (1976). *Fairness in Social Relationships.* Morristown, NJ: General Learning Press.

[B71] Lian H. Ferris D. L. Brown D. J. (2012). Does power distance exacerbate or mitigate the effects of abusive supervision? it depends on the outcome. *J. Appl. Psychol.* 97 107–123. 10.1037/a0024610 21766996

[B72] Liden R. C. Mitchell T. R. (1988). Ingratiatory behaviors in organizational settings. *Acad. Manag. Rev.* 13 572–587. 10.5465/amr.1988.4307430

[B73] Lin S. H. Scott B. A. Matta F. K. (2019). The dark side of transformational leader behaviors for leaders themselves: a conservation of resources perspective. *Acad. Manag. J.* 62 1556–1582. 10.5465/amj.2016.1255

[B74] Lin W. Wang L. Chen S. (2013). Abusive supervision and employee well-being: the moderating effect of power distance orientation. *Appl. Psychol.* 62 308–329. 10.1111/j.1464-0597.2012.00520.x

[B75] Loi R. Lam L. W. Chan K. W. (2012). Coping with job insecurity: the role of procedural justice, ethical leadership and power distance orientation. *J. Bus. Ethics* 108 361–372. 10.1007/s10551-011-1095-3

[B76] Lv Q. Xu S. Ji H. (2012). Emotional labor strategies, emotional exhaustion, and turnover intention: an empirical study of chinese hotel employees. *J. Hum. Resour. Hos. Tour.* 11 87–105. 10.1080/15332845.2012.648837

[B77] Marcus B. Schuler H. (2004). Antecedents of counterproductive behavior at work: a general perspective. *J. Appl. Psychol.* 89 647–660. 10.1037/0021-9010.89.4.647 15327351

[B78] Martinko M. J. Gundlach M. J. Douglas S. C. (2002). Toward an integrative theory of counterproductive workplace behavior: a causal reasoning perspective. *Int. J. Select. Assess.* 10 36–50. 10.1111/1468-2389.00192

[B79] Maslach C. Jackson S. E. (1981). The measurement of experienced burnout. *J. Organ. Behav.* 2 99–113. 10.1002/job.4030020205

[B80] Maslach C. Schaufeli W. B. Leiter M. P. (2001). Job burnout. *Annu. Rev. Psychol.* 52 397–422. 11148311 10.1146/annurev.psych.52.1.397

[B81] Masterson S. S. Lewis K. Goldman B. M. Taylor M. S. (2000). Integrating justice and social exchange: the differing effects of fair procedures and treatment on work relationships. *Acad. Manag. J.* 43 738–748. 10.5465/1556364

[B82] Meydan C. H. Basim N. H. Başar U. (2014). Power distance as a moderator of the relationship between organizational citizenship behavior and impression management. *Eurasian J. Bus. Econ.* 7 105–118.

[B83] Mikula G. Scherer K. R. Athenstaedt U. (1998). The role of injustice in the elicitation of differential emotional reactions. *Pers. Soc. Psychol. Bull.* 24 769–783. 10.1177/0146167298247009

[B84] Moss S. E. Valenzi E. R. Taggart W. (2003). Are you hiding from your boss? The development of a taxonomy and instrument to assess the feedback management behaviors of good and bad performers. *J. Manag.* 29 487–510. 10.1016/s0149-2063(03)00022-9

[B85] Park S. H. Westphal J. D. Stern I. (2011). Set up for a fall: the insidious effects of flattery and opinion conformity toward corporate leaders. *Admin. Sci. Q.* 56 257–302. 10.1177/0001839211429102

[B86] Penney L. M. Hunter E. M. Perry S. J. (2011). Personality and counterproductive work behavior: using conservation of resources theory to narrow the profile of deviant employees. *J. Occup. Organ. Psychol.* 84 58–77. 10.1111/j.2044-8325.2010.02007.x

[B87] Pines A. Maslach C. (1978). Characteristics of staff burnout in mental health settings. *Psychiatr.Serv.* 29 233–237. 10.1176/ps.29.4.233 631745

[B88] Preacher K. J. Hayes A. F. (2008). Asymptotic and resampling strategies for assessing and comparing indirect effects in multiple mediator models. *Behav. Res. Methods* 40 879–891. 10.3758/brm.40.3.879 18697684

[B89] Reh S. Tröster C. Van Quaquebeke N. (2018). Keeping (Future) rivals down: temporal social comparison predicts coworker social undermining via future status threat and envy. *J. Appl. Psychol.* 103 399–415. 10.1037/apl0000281 29239645

[B90] Roberge M. É Van Dick R. (2010). Recognizing the benefits of diversity: when and how does diversity increase group performance? *Hum. Resour. Manag. Rev.* 20 295–308. 10.1016/j.hrmr.2009.09.002

[B91] Robinson S. L. Bennett R. J. (1995). A typology of deviant workplace behaviors: a multidimensional scaling Study. *Acad. Manag. J.* 38 555–572. 10.5465/256693

[B92] Rorty A. O. (2000). The dramas of resentment. *Yale Rev.* 88 89–100. 10.1111/0044-0124.00417

[B93] Sapolsky R. M. (2004). *Why Zebras don’t Get Ulcers: The Acclaimed Guide to Stress, Stress-related Diseases, and Coping-now Revised and Updated.* New York, NY: Holt Paperbacks.

[B94] Schaufeli W. B. Bakker A. B. (2004). Job demands, job resources, and their relationship with burnout and engagement: a multi-sample Study. *J. Organ. Behav.* 25 293–315. 10.1002/job.248

[B95] Schaufeli W. B. Dierendonck D. V. Gorp K. V. (1996). Burnout and reciprocity: towards a dual-level social exchange model. *Work Stress* 10 225–237. 10.1080/02678379608256802

[B96] Schaufeli W. B. Leiter M. P. Maslach C. (2009). Burnout: 35 years of research and practice. *Eng. Manag. Rev. IEEE* 38 4–18. 10.1109/emr.2010.5645750

[B97] Shropshire C. (2010). The role of the interlocking director and board receptivity in the diffusion of practices. *Acad. Manag. Rev.* 35 246–264. 10.5465/amr.2010.48463333

[B98] Smith R. H. Kim S. H. (2007). Comprehending Envy. *Psychol. Bull.* 133 46–64. 10.1037/0033-2909.133.1.46 17201570

[B99] Sonnentag S. Frese M. (2003). *Stress in Organizations. Comprehensive Handbook of Psychology: Industrial and Organizational Psychology.* New York, NY: Wiley.

[B100] Spector P. E. Fox S. (2005). “The Sressor-emotion Model of Counterproductive Work Behavior,” in *Counterproductive Work Behavior: Investigations of Actors and Targets*, eds Fox S. Spector P. E. (Washington, DC: American Psychological Association).

[B101] Spector P. E. Fox S. (2010). Counterproductive work behavior and organizational citizenship behavior: are they opposite forms of active behavior? *Appl. Psychol.* 59 21–39. 10.1111/j.1464-0597.2009.00414.x 20604597

[B102] Spector P. E. Fox S. Penney L. M. Bruursema K. Goh A. Kessler S. (2006). The dimensionality of counterproductivity: are all counterproductive behaviors created equal? *J. Vocat. Behav.* 68 446–460. 10.1016/j.jvb.2005.10.005

[B103] Taris T. W. Schreurs P. J. Van Iersel-Van Silfhout I. J. (2001). Job stress, job strain, and psychological withdrawal among dutch university staff: towards a dual process model for the effects of occupational stress. *Work Stress* 15 283–296. 10.1080/02678370110084049

[B104] Tedeschi J. T. Melburg V. (1984). Impression management and influence in the organization. *Res. Sociol. Organ.* 3 31–58.

[B105] Tepper B. J. Moss S. E. Lockhart D. E. Carr J. C. (2007). Abusive supervision, upward maintenance communication, and subordinates’ psychological distress. *Acad. Manag. J.* 50 1169–1180. 10.2307/20159918

[B106] Thorisdottir H. Jost J. T. Kay A. C. (2009). “Social and psychological bases of ideology and system justification,” in *Social and Psychological Bases of Ideology and System Justification*, eds Jost J. T. Kay A. C. Thorisdottir H. (Oxford: Oxford University Press).

[B107] Treadway D. C. Ferris G. R. Duke A. B. Adams G. L. Thatcher J. B. (2007). The moderating role of subordinate political skill on supervisors’ impressions of subordinate ingratiation and ratings of subordinate interpersonal facilitation. *J. Appl. Psychol.* 92 848–855. 10.1037/0021-9010.92.3.848 17484564

[B108] Turnley W. H. Bolino M. C. (2001). Achieving desired images while avoiding undesired images: exploring the role of self-monitoring in impression management. *J. Appl. Psychol.* 86 351–360. 10.1037/0021-9010.86.2.351 11393446

[B109] Tyler T. R. Lind E. A. Huo Y. J. (2000). Cultural values and authority relations: the psychology of conflict resolution across cultures. *Psychol. Public Policy Law* 6 1138–1163. 10.1037/1076-8971.6.4.1138

[B110] Vohs K. D. Baumeister R. F. (2004). *Understanding Self-regulation: An Introduction. Handbook of Self-regulation: Research, Theory, and Applications.* New York, NY: Guilford Press.

[B111] Vohs K. D. Baumeister R. F. Ciarocco N. J. (2005). Self-regulation and self-presentation: regulatory resource depletion impairs impression management and effortful self-presentation depletes regulatory resources. *J. Pers. Soc. Psychol.* 88 632–657. 10.1037/0022-3514.88.4.632 15796665

[B112] Watson D. Clark L. A. Tellegen A. (1988). Development and validation of brief measures of positive and negative affect: the panas scales. *J. Pers. Soc. Psychol.* 54 1063–1070. 10.1037/0022-3514.54.6.1063 3397865

[B113] Welsh D. T. Baer M. D. Sessions H. (2020). Hot pursuit: the affective consequences of organization-set versus self-set goals for emotional exhaustion and citizenship behavior. *J. Appl. Psychol.* 105 166–185. 10.1037/apl0000429 31219258

[B114] Westphal J. D. Stern I. (2006). The other pathway to the boardroom: interpersonal influence behavior as a substitute for elite credentials and majority status in obtaining board appointments. *Admin. Sci. Q.* 51 169–204. 10.2189/asqu.51.2.169 21821037

[B115] Whitman M. V. Halbesleben J. R. Holmes O. IV (2014). Abusive supervision and feedback avoidance: the mediating role of emotional exhaustion. *J. Organ. Behav.* 35 38–53. 10.1002/job.1852

[B116] Wright T. A. Cropanzano R. (1998). Emotional exhaustion as a predictor of job performance and voluntary turnover. *J. Appl. Psychol.* 83 486–493. 10.1037/0021-9010.83.3.486 9648526

[B117] Wright T. A. Hobfoll S. E. (2004). Commitment, psychological well-being and job performance: an examination of conservation of resources (COR) theory and job burnout. *J. Bus. Manag.* 9 389–403.

[B118] Yam K. C. Chen X. P. Reynolds S. J. (2014). Ego depletion and its paradoxical effects on ethical decision making. *Organ. Behav. Hum. Dec. Process.* 124 204–214. 10.1016/j.obhdp.2014.03.008

